# Impact of Potential Blockers on Ru(III) Complex Binding to Human Serum Albumin

**DOI:** 10.1155/S1565363303000116

**Published:** 2003

**Authors:** Lilianna Trynda-Lemiesz, Marek Łuczkowski

**Affiliations:** Faculty of Chemistry University of Wroclaw F. Joliot-Curie 14 Wroclaw 50-383 Poland

## Abstract

The effects of aspirin, vitamin B2 and warfarin as potential blockers of the ruthenium binding sites in HSA were investigated through UV/visible, circular dichroism (CD), fluorescence spectroscopy and the inductively coupled plasma-atomic emission spectroscopy ICP(AES). The studies on the interactions of several biologically relevant molecules with HSA have shown that drugs like aspirin or warfarin may strongly influence the interaction of serum protein with anticancer drugs. It can derive from the influence of the drug on protein conformation or binding close to binding site of anticancer drug. Aspirin, vitB2 and warfarin bind to IIA subdomain leading to partial blocking of the ruthenium binding site in HSA.


### Impact of Potential Blockers on Ru(III) Complex Binding to Human Serum Albumin

Lilianna Trynda-Lemiesz,* and Marek Łuczkowski

*Faculty of Chemistry, University of Wroclaw, F. Joliot-Curie 14, 50-383 Wroclaw, Poland*
*Email:LTL@wchuwr.chem.uni.wroc.pl*

(Received February 11, 2003; Revision March 17, 2003; Accepted March 18, 2003)

### ABSTRACT

The effects of aspirin, vitamin $B_{12}$ and warfarin as potential blockers of the ruthenium binding sites in HSA were investigated through UV/visible, circular dichroism (CD), fluorescence spectroscopy and the inductively coupled plasma-atomic emission spectroscopy ICP(AES). The studies on the interactions of several biologically relevant molecules with HSA have shown that drugs like aspirin or warfarin may strongly influence the interaction of serum protein with anticancer drugs. It can derive from the influence of the drug on protein conformation or binding close to binding site of anticancer drug. Aspirin, vit$B_{12}$ and warfarin bind to IIA subdomain leading to partial blocking of the ruthenium binding site in HSA.

**Keywords:** Ruthenium(III); Human Serum Albumin; Circular dichroism, Fluorescence quenching; Aspirin; Warfarin; Vitamin $B_{12}$

### INTRODUCTION

A full understanding of the modes of action of the metal-based antitumoral drugs requires the detailed study of their interactions with all possible biological targets including amino acids, hormones, peptides and

---

* All correspondence to Dr. Lilianna Trynda-Lemiesz
Faculty of Chemistry, University of Wrocław
F.Joliot-Curie 14
50-383 Wrocław, Poland
fax +48 71 3282348
Email: LTL@wchuwr.chem.uni.wroc.pl proteins. Albumin is the most abundant plasma protein and the interaction with metal anticancer drugs may have a major influence on drug pharmacology and efficacy.

A number of Ru(III) complexes exhibit antitumor activities in animal models /1/. Two anticancer ruthenium (III) complexes, with imidazole and indazole ligands are highly active compared to other ruthenium(III) compounds. Both of these complexes show excellent antitumor activity in various animal models /2/. The most promising complex is HInd[$RuInd_2Cl_4$] – abbrev. Ru-ind (scheme 1) – which is far less toxic in long term application than the imidazole analogue /3/. This has inspired considerable interest in the study of the biochemical behavior of the Ru-ind compounds including their interactions with serum proteins, which are the primary target molecules, when metal compound is administered intravenously /4/. The preferred binding sites for the Ru(III) complexes are at histidyl residues of the protein, presumably following the loss of one or more of the chloride ligands /5/. The major amount of ruthenium species (80-90 %) is bound to albumin and a much smaller amount to transferrin /4/. Apotransferrin may act as a natural carrier of the ruthenium drug and this has caused strong interest in the studies on Ru(III)-apotransferrin system /6, 7/. The coordination of ruthenium to apotransferrin can be used to enhance tumor uptake, since neoplastic cells, especially those in rapidly growing tumors, have a high iron requirement and display a large number of transferrin receptors on their membrane surfaces. Furthermore, it has been demonstrated that the apotransferrin-bound Ru-ind complex exhibits a significantly higher antitumor activity against human colon cancer cells when compared with the "free" species /4/. In contrast ruthenium-albumin bound species have no significant antitumor activity.

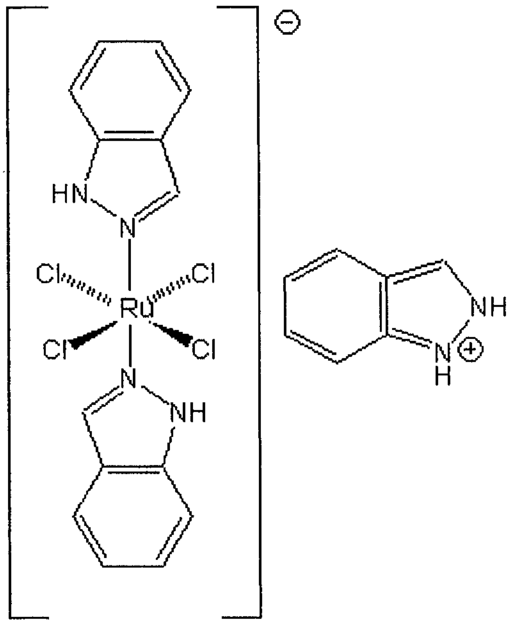

**Scheme 1:** Schematic representation of HInd[$RuInd_2Cl_4$] complex

Interactions with albumin could be of critical importance for understanding the Ru(III) toxicity and its distribution within the organism. This metal-species binding would lead to altered protein conformation and changes in biological activity of the protein /8, 9/. Human serum albumin (HSA), is a single-chain 66 kDa protein, which is largely α-helical, and consists of three structurally homologous domains that assemble to form a heart-shaped molecule. Each domain is a product of two subdomains, which are predominantly helical and extensively cross-linked by several disulfide bridges /10/. Its amino-acid sequence contains a total of 17 disulphide bridges, one free thiol (Cys 34) and a single tryptophan (Trp 214). Albumin is known to bind and transport many ligands, including fatty acids, amino acids, steroids, metal ions, and a variety of pharmaceuticals /11, 12/. The principal regions of ligand binding sites of albumin are located in hydrophobic cavities in subdomains IIA and IIIA, which exhibit similar chemistry. The binding locations have been determined crystallographically for several ligands. The IIIA subdomain is the most active in accommodating many ligands, as for example, digitoxin, ibuprofen and tryptophan. Aspirin show nearly equal distributions between binding sites located in IIA and III A subdomains, while warfarin occupies a single site in IIA /12/. The high-affinity site for bilirubin has been isolated in domain II /13/, and the principal binding site for long-chain fatty acids has been shown to occupy the domain III /14/. The likely location of the multi-metal site in human albumins is thus the area of contact of domains I and II /15/. Aquacobalamin (aqua-$B_{12}$) binds to albumin via imidazole groups of histidyl residues located in IIA and IIIA subdomain /16/.

Ligand binding to one domain induces distinct conformational changes in the other domain, as both subdomains share a common interface. Thus, the binding of particular drug molecule to serum albumin may change considerably binding abilities of HSA towards other molecules. A previous study /9/ has shown that albumin can specifically bind up to four moles of the Ru-indazole complex. In this work the effects of aspirin, vitamin -$B_{12}$ and warfarin as potential blockers of the ruthenium binding sites in HSA were investigated.

### EXPERIMENTAL

### Materials

Human serum albumin and sodium salicylate were obtained from Fluka Chem.Co. Aspirin, warfarin, Sephadex G-25 and apo-transferrin were purchased from Sigma, aquo-$B_{12}$ from Serva. HSA concentration was determined by absorption spectrum, taking the absorbance of a 1mg/$cm^3$ solution at 280 nm as 0.55 /17/. Aspirin acetylated HSA was prepared according to the procedure of Pincard *et al.* /18/ Trans-indazolium bisindazole-tetrachlororuthenate(III) was synthesized as described earlier /19/ and was used in all experiments from a freshly prepared $5x10^{-4}$M aqueous solution. In all the experiments a physiological buffer was used so that the final concentrations were 0.004 M $NaH_2PO_4$, 0.1M NaCl and 0.025M $NaHCO_3$, pH 7.4.

### Methods

Absorption spectra were recorded on a BECKMAN DU-650 spectrophotometer, and CD spectra on a JASCO J-715 spectropolarimeter. CD spectra were recorded over the range of 200-250 and 250-360 nm, 300-600 nm, using 0.1 and 1.0 cm cells respectively. Fluorescence measurements were carried out on an

SLM AMINco SPF-500 spectrofluorimeter with the excitation and emission wavelength set at 335 and 378 nm (warfarin).

The assays of ruthenium bounded per mol of HSA were performed with a Spectrometer 3410 (Inductively Couplet Plasma-Atomic Emission Spectroscopy, ICP-AES). The protein fractions bound with ruthenium complex were separated by gel-filtration chromatography on Sephadex G-25 column. The ruthenium content in the selection fraction was determined by the ICP method.

### RESULTS AND DISCUSSION

Binding of Ru(III) indazole species to albumin has a strong impact on protein structure and physiological functions, but essentially this interaction may have a major influence on drug pharmacology and efficacy and plays an important role in the metabolism of this anticancer agent.

Transferrin may act as a moderately selective carrier for HInd[$RuInd_2Cl_4$] complex into tumor cells. Ru-Tf has a substantially greater uptake into tumors, probably because of the unusually large number of transferrin receptors on the surface of cancer cells. The potential of using transferrin conjugates for selective drug delivery to tumor tissue should be seen in light of the natural transferrin cycle and iron metabolism. Structural differences between transferrin, apotransferrin and Ru(III)-apotransferrin (Fig. 1) are very small and for that reason, transferrin might be able to act as a selective drug delivery system for antitumor ruthenium complexes.

The albumin structure (Fig. 2) /20/ is predominantly α-helical. Approximately, 67% of HSA is helical, the number of helices in the structure is 28 /12/. CD spectra of HSA exhibit two negative bands in the ultraviolet region at 209 and 220 nm characteristic for an α-helical structure of protein. The binding of ruthenium complex to HSA decreases both of these bands /9/. This clearly indicates the changes in the protein secondary structure.

### Aspirin acetylated albumin

The two lysine residues on albumin are highly reactive toward tribenzenosulfonates and aspirin. At low aspirin concentrations, a single lysine residue of HSA is selectively acetylated by aspirin. Walker /21/ has identified the predominant site of acetylation as lysine 199. The binding locations determined crystallographically /12/ indicated that aspirin shows nearly equal distributions between binding sites located in IIA and III A subdomains. CD spectroscopy measurements show (Fig. 3), that acetylation of albumin at low aspirin concentrations results in marked conformational changes of the protein. Further conformational changes in the HSA structure induced by ruthenium coordination indicate that aspirin is not able to prevent ruthenium binding. However, it may induce some conformational changes and local perturbations at the ruthenium binding sites.

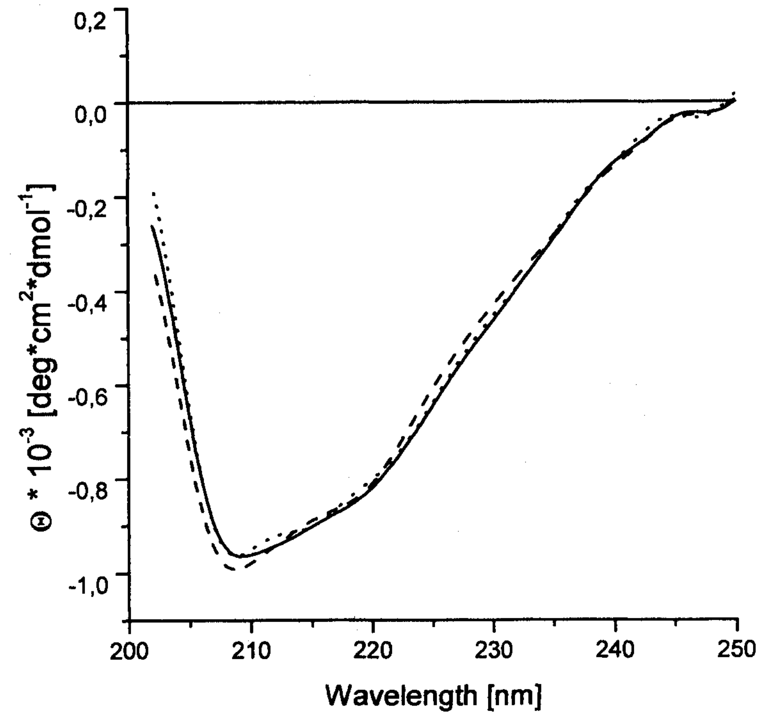

**Fig. 1:** UV CD spectra of native transferrin (solid line), native apotransferrin (dashed line) and native apotransferrin incubated with $HInd[RuInd_2Cl_4]$ complex (dotted line) after 24 hours of reaction running at 37°C. $c_{apotransferrin} = 2 * 10^{-6}$ M; molar ratio Ru(III)/apotransferrin = 2:1

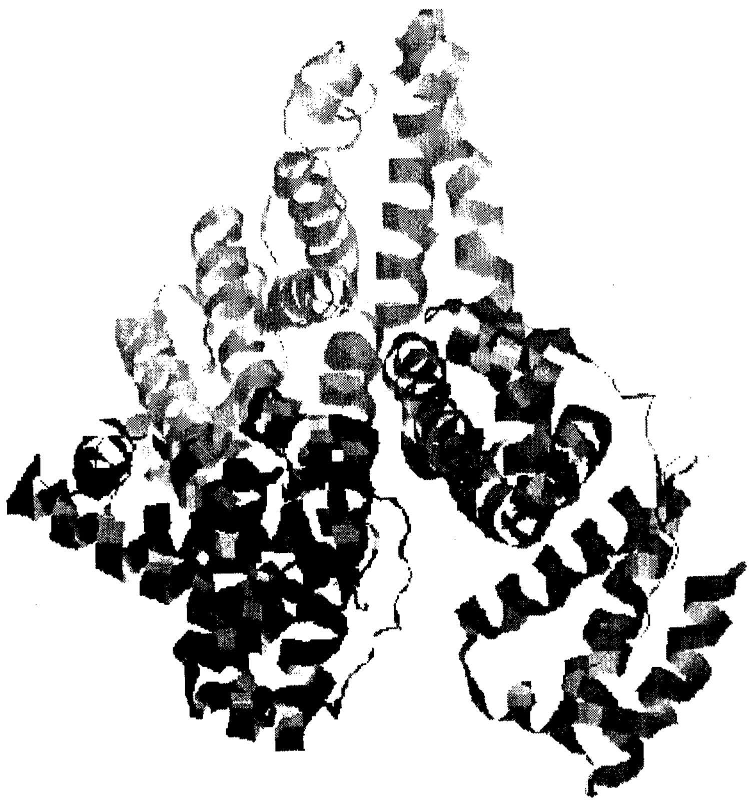

**Fig. 2:** Crystal structure of Human Serum Albumin /20/.

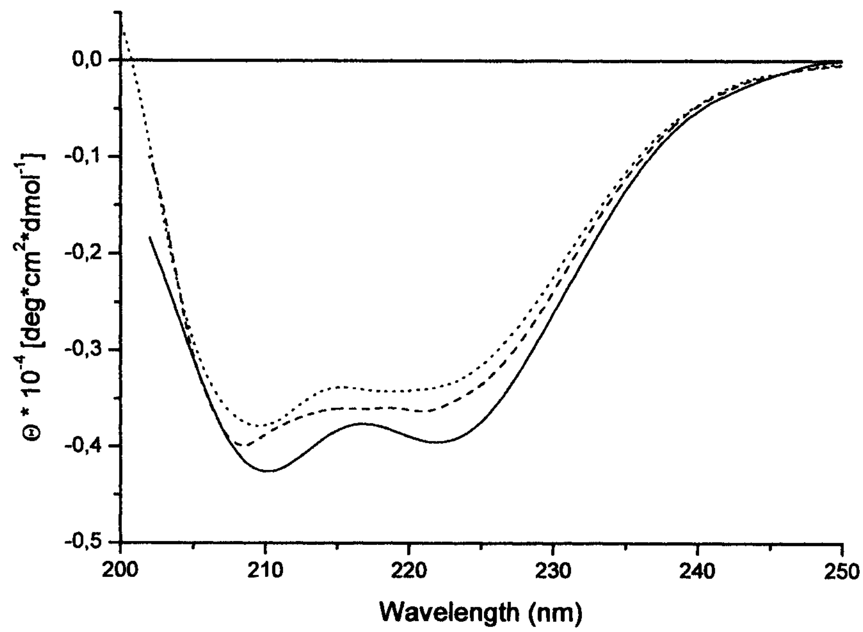

**Fig. 3:** UV CD spectra of native HSA (solid line), acetylated HSA (dashed line) and acetylated HSA incubated with $HInd[RuInd_2Cl_4]$ complex (dotted line) after 24 hours of reaction running at 37°C. $c_{Ac\text{-}HSA} = 8 * 10^{-6}$ M, $c_{Ru\text{-}ind} = 1.6 * 10^{-5}$ M

### Warfarin

*Warfarin* is one of the best characterized drugs as far as the binding sites in HSA are concerned /22/. Site I is located in subdomain IIA near Trp-214 /23/. Warfarin has a weak fluorescence at 378 nm when excited at 335 nm, and the addition of HSA induced an increase in fluorescence intensity when warfarin binds to a single site in the protein /24/. Binding of ruthenium(III) complexes to HSA has an influence on warfarin binding site in albumin /9/. $HInd[RuInd_2Cl_4]$ causes sevenfold decrease of a relative fluorescence intensity at 335 nm. However, the interaction of ruthenium compound with albumin already preincubated with warfarin quenches only slightly the intensity of this band (Fig. 4). This may suggest that $HInd[RuInd_2Cl_4]$ and warfarin compete for the binding sites located at $Trp_{214}$ residue and that binding of warfarin to HSA protects the binding of $HInd[RuInd_2Cl_4]$ to the protein.

The changes in the CD spectra (Fig. 5) confirm the results achieved in fluorescence measurements. The characteristic shape of the spectra for HSA preincubated with $HInd/RuInd_2Cl_4/$ is distinctly different from that of the native HSA, while the shape of the spectra obtained for HSA incubated prior with warfarin and then with ruthenium compound is almost the same although with decreased bands intensities.

### Aquacobalamin (aqua-$B_{12}$)

16.5% of the serum $B_{12}$ is bound to albumin via imidazole groups of histidyl residues located in IIA and IIIA subdomain /25/. Binding of aquacobalamin to HSA can be easily monitored by spectroscopic methods, especially the CD spectra. The CD spectra of aqua-form of vitamin $B_{12}$ bound to HSA differ considerably

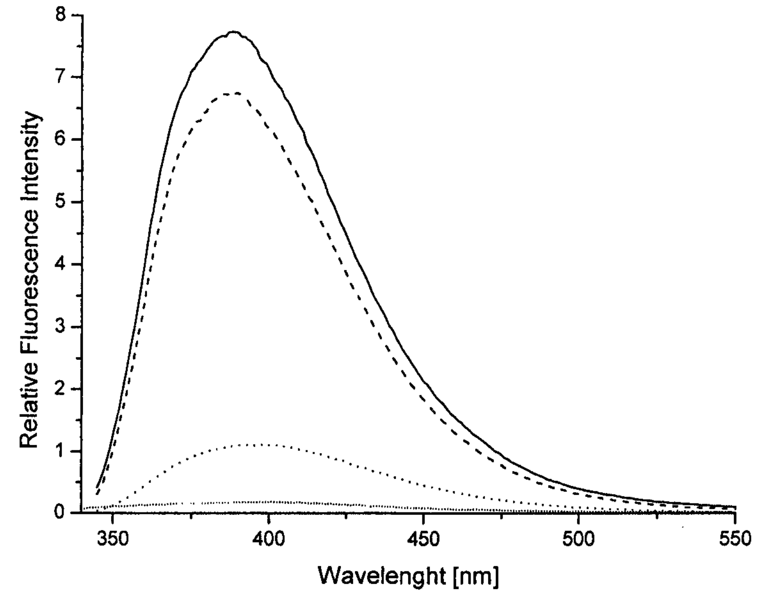

**Fig. 4:** Relative fluorescence intensity changes of warfarin with ruthenium treated HSA after 24 hours of reaction running at 37°C. HSA-warfarin (solid line), HSA-warfarin+ $HInd[RuInd_2Cl_4]$ (dashed line), HSA-$HInd[RuInd_2Cl_4]$+warfarin (dotted line), warfarin (short-dotted line); $c_{HSA} = 4* 10^{-5}$ M, $c_{warfarin} = 8* 10^{-5}$ M, molar ratio Ru(III)/HSA = 2:1 excitation $\lambda = 335$ nm, emission $\lambda = 378$ nm

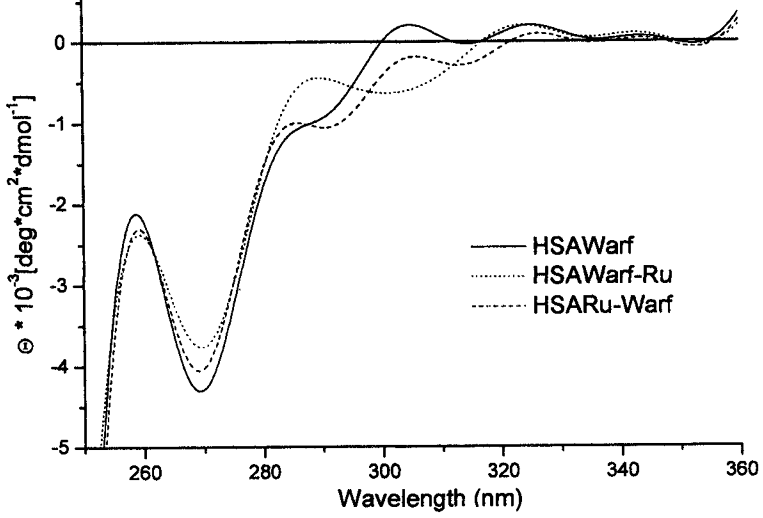

**Fig. 5:** Effect of Ru(III)-indazole on the visible CD spectrum of HSA-warfarin. HSA-warfarin (solid line), HSA-warfarin+ $HInd[RuInd_2Cl_4]$ (dashed line), HSA-$HInd[RuInd_2Cl_4]$+warfarin (dotted line); $c_{HSA} = 4* 10^{-5}$ M, $c_{warfarin} = 8* 10^{-5}$ M, molar ratio Ru(III)/HSA = 2:1

from those of the free vitamin (see Fig. 6). The band intensities increase and the single band at 483 nm appears as a consequence of the complex formation. $HInd[RuInd_2Cl_4]$ may influence aquacobalamin binding by partial blocking of its binding site or inducing conformational changes leading to decreased affinity of HSA to the vitamin.

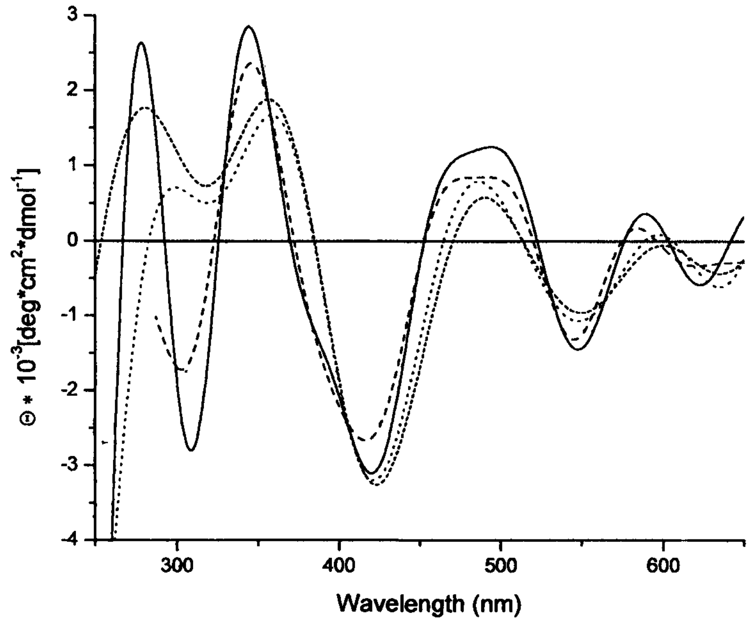

**Fig. 6:** Effect of Ru(III)-indazole on the visible CD spectrum of HSA-$B_{12}$. HSA-$B_{12}$ (solid line), HSA-$B_{12}$+ HInd[$RuInd_2Cl_4$] (dashed line), HSA-HInd[$RuInd_2Cl_4$]+$B_{12}$ (dotted line), $B_{12}$ (short-dotted line); $c_{HSA} = 4* 10^{-5}$ M, $c_{B12} = 8* 10^{-5}$ M, molar ratio Ru(III)/HSA = 2:1

### CONCLUSIONS

The interaction of anticancer drugs with blood constituents, particularly with serum albumin may have a major influence on drug pharmacology and efficacy /26/.

As already shown /4/, about 80% of ruthenium complexes bind to HSA and a much smaller amount to transferrin. It has been demonstrated that the apotransrerrin-bound ruthenium complex exhibits a significantly higher antitumor activity against human colon cancer cells when compared with the “free” species. In contrast ruthenium-albumin bound species have no significant antitumor activity.

It is known that the binding of a particular drug molecule to serum albumin may change considerably the binding abilities of HSA towards other molecules. The relatively neutral drugs such as aspirin or vitamin $B_{12}$ and warfarin, a widely used anticoagulant, which is 99% bound to protein, bind to IIA subdomain in HSA leading to partial blocking of ruthenium binding site. A previous study /9/ has shown that native albumin can specifically bind up to four moles of the Ru(III)-indazole complex. After modification of HSA by warfarin, vit$B_{12}$, and aspirin the ruthenium content decreases to 1.6, 1.65 and 1.2 moles per mol of the protein, respectively (Fig.7). Warfarin, vit$B_{12}$ and aspirin may influence ruthenium binding by partial blocking of its binding site or inducing conformational changes leading to decreased affinity of HSA to the Ru(III)-indazole complex.

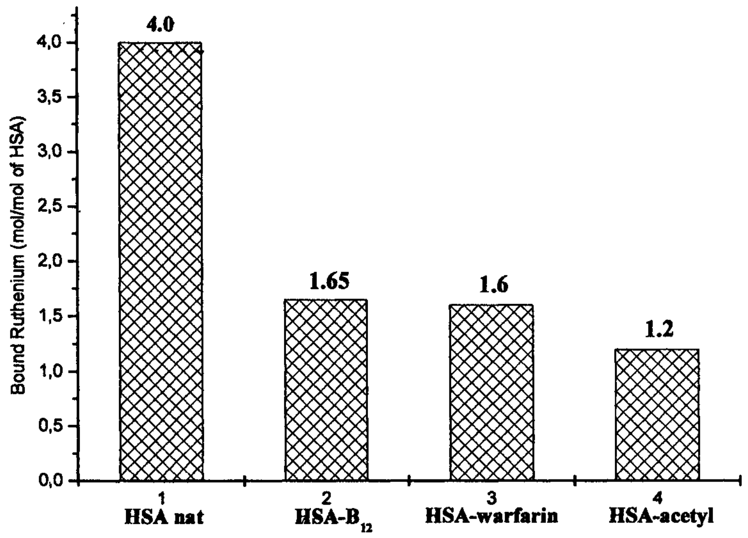

**Fig. 7:** The binding of ruthenium complex to HSA, after modification by warfarin, vitamin $B_{12}$ and aspirin. The protein fractions bound with ruthenium were separated by gel-filtration chromatography. Ru contents in selection fractions were determined using the ICP(AES) method.

### ACKNOWLEDGEMENTS

This work was supported by the Polish State Committee for Scientific Research (KBN 3 P05F 054 22). This work was performed within COST D20 project.

### REFERENCES

1. M.J. Clarke, Ruthenium chemistry pertaining to the design of anticancer agents, In: *Progress in Clinical Biochemistry and Medicine*, Vol.10, Springer-Verlag, Berlin Heidelberg, 1989; pp. 25-39.
2. B.K. Keppler, G. Lipponer, B. Stenzel and F. Kranz, In: *Metal Complexes in Cancer Chemotherapy*, B.K. Keppler ed., VCH, Weinheim, (1993), pp.187-220.
3. B.K. Keppler and M. Hartmann, *Metal Based Drugs*, **1**, 145 (1994).
4. F. Kratz, N. Mulinacci, L. Messori, I. Bertini and B.K. Keppler, *Metal Ions in Biology and Medicine*, J. Anastassopoulou, Ph. Collery, J.C. Etienne and Th. Theophanides, eds., J.Libbey Eurotext, Paris, vol.2, (1992), pp. 69-74.
5. F. Kratz, B.K. Keppler, L. Messori, C. Smith and E.N. Baker, *Metal Based Drugs*, **1**, 169 (1994).
6. F. Kratz, M. Hartmann, B.K. Keppler and L. Messori, *J. Biol. Chem.*, **269**, 2581 (1994).
7. F. Kratz and L. Messori, *J. Inorg. Biochem.*, **49**, 79 (1993).
8. L. Trynda-Lemiesz, B.K. Keppler and H. Kozłowski, *J. Inorg. Biochem.*, **73**, 123 (1999).
9. L. Trynda-Lemiesz, A. Karaczyn B.K. Keppler and H. Kozłowski, *J. Inorg. Biochem.*, **78**, 341(2000).
10. D.C. Carter and J.X. Ho, *Adv. Protein Chem.*, **45**, 153 (1994).

11. T. Peters, Jr., *Adv. Protein Chem.*, **37**, 161 (1985).
12. X.M. He and D.C. Carter, *Nature*, **358**, 209 (1992).
13. G. Sudlow, D.J. Birkett and D.N. Wade, *Mol. Pharmacol.*, **12**, 1052 (1976).
14. B.J. Soltys and J.C. Hsia, *J. Biol. Chem.*, **253**, 3023 (1978).
15. P.J. Sadler and J. Viles, *Inorg. Chem.*, **35**, 4490 (1996).
16. R.T. Taylor and M.L. Hanna, *Arch. Bioch. Biophys.*, **141**, 247 (1970).
17. G.H. Beaven, S.H. Chen, A d'Albis and W.B. Gratzer, *Eur. J. Bioch.*, **41**, 539 (1974).
18. R.N. Pinkard, D. Hawkins and R.S. Fart, *Arthritis Rheum.*, **31**, 361 (1975).
19. B.K. Keppler, M. Henn, U.M. Juhl, M.R. Berger, R. Niebl and F.E. Wagner, *Prog. Clin. Bioch. Med.*, **10**, 41 (1989).
20. S. Sugio, A. Kashima, S. Mochizuki, M. Noda and K. Kobayashi, *Protein Eng.*, **12**, 439 (1999).
21. J.E.Walker, *FEBS Lett.*, **66**, 173 (1976).
22. O.J.M. Bos, J.P.M. Remijn, J.E. Fisher, J.Witing and L.H.M. Janssen, *Biochem Pharmac.*, **37**, 3905 (1988).
23. I. Petitpas, A.A Bhattacharya, S. Twine, M. East and S. Curry, *J. Biol. Chem.*, **276**, 22804 (2001).
24. K.J. Fehske, W.E. Muller and U. Wollert, *Mol. Pharmacol.*, **16**, 778 (1979).
25. R.T. Taylor and M.L. Hanna, *Arch. Bioch. Biophys.*, **141**, 247 (1970).
26. L. Takahashi, T.Ohnuma, S. Kavy, S. Bhardwaj and J.F.Holland, *Br. J. Cancer*, **41**, 602 (1980).

